# Advancements in Rift Valley fever vaccines: a historical overview and prospects for next generation candidates

**DOI:** 10.1038/s41541-023-00769-w

**Published:** 2023-11-04

**Authors:** Cigdem Alkan, Eduardo Jurado-Cobena, Tetsuro Ikegami

**Affiliations:** 1 Department of Pathology, The University of Texas Medical Branch at Galveston, 301 University Blvd, Galveston, TX 77555 USA; 2 Department of Microbiology and Immunology, The University of Texas Medical Branch at Galveston, 301 University Blvd, Galveston, TX 77555 USA; 3 The Sealy Institute for Vaccine Sciences, The University of Texas Medical Branch at Galveston, 301 University Blvd, Galveston, TX 77555 USA; 4 The Center for Biodefense and Emerging Infectious Diseases, The University of Texas Medical Branch at Galveston, 301 University Blvd, Galveston, TX 77555 USA

**Keywords:** Live attenuated vaccines, Translational research

## Abstract

Rift Valley fever (RVF) is a zoonotic viral disease transmitted by mosquitoes and causes abortion storms, fetal malformations, and newborn animal deaths in livestock ruminants. In humans, RVF can manifest as hemorrhagic fever, encephalitis, or retinitis. Outbreaks of RVF have been occurring in Africa since the early 20th century and continue to pose a threat to both humans and animals in various regions such as Africa, Madagascar, the Comoros, Saudi Arabia, and Yemen. The development of RVF vaccines is crucial in preventing mortality and morbidity and reducing the spread of the virus. While several veterinary vaccines have been licensed in endemic countries, there are currently no licensed RVF vaccines for human use. This review provides an overview of the existing RVF vaccines, as well as potential candidates for future studies on RVF vaccine development, including next-generation vaccines that show promise in combating the disease in both humans and animals.

## Introduction

Rift Valley fever (RVF) is a viral disease that poses a significant threat to public health and the livestock industry in various regions, including sub-Saharan Africa, the Comoros, Madagascar, Egypt, Saudi Arabia, and Yemen^1–3^. This zoonotic disease is transmitted by mosquitoes and affects both humans and ruminants. The virus responsible for RVF, known as Rift Valley fever virus (RVFV), relies on various mosquito species to amplify and spread the infection through the ingestion of blood meals from viremic animals^2^. Furthermore, RVFV has demonstrated the ability to establish vertical viral transmission in floodwater *Aedes spp*. mosquitoes, enabling the virus to survive for extended periods in dehydrated eggs and contributing to longer inter-epizootic periods^4^. Recent laboratory studies have even shown the potential for vertical viral transmission in *Culex tarsalis* mosquitoes, suggesting a possible role for *Culex* spp. mosquitoes in the persistence of RVFV in endemic regions^5^. Despite sporadic major outbreaks, RVF remains a significant public health concern and inflicts severe economic damage on the livestock industry. Recognizing its impact, the World Health Organization designates RVF as a priority human disease, and the World Organisation for Animal Health classifies it as a notifiable disease. Moreover, RVFV is classified as a Category A Priority Pathogen by the National Institute of Allergy and Infectious Disease and an overlap select agent by the United States Department of Health and Human Services and Agriculture^6,7^. To address the ongoing threat of RVF, extensive research has been conducted on existing RVF vaccines and potential candidates for future vaccine development. This review aims to provide an overview of the current RVF vaccines and explore next-generation vaccines that show promise in combating the disease in both humans and animals. By understanding the landscape of RVF vaccine research, we can identify strategies to mitigate the impact of this devastating viral disease.

## Outbreak history of Rift Valley fever

RVF outbreaks are characterized by high rates of spontaneous abortion, also known as “abortion storm,” in sheep, goats, and cattle, often preceding the onset of the outbreak. Newborn lambs and goat kids are particularly vulnerable to acute RVF infections, and mortality is common^3^. In humans, the disease can be contracted through direct contact with bodily fluids from infected animals or mosquito bites, with most patients experiencing a self-limiting febrile illness^8^. However, up to 8% of patients may develop more severe symptoms, such as hemorrhagic fever, encephalitis, or retinitis, associated with partial or complete blindness and a mortality rate of 0.5 to 1.0%.

**Fig. 1:**
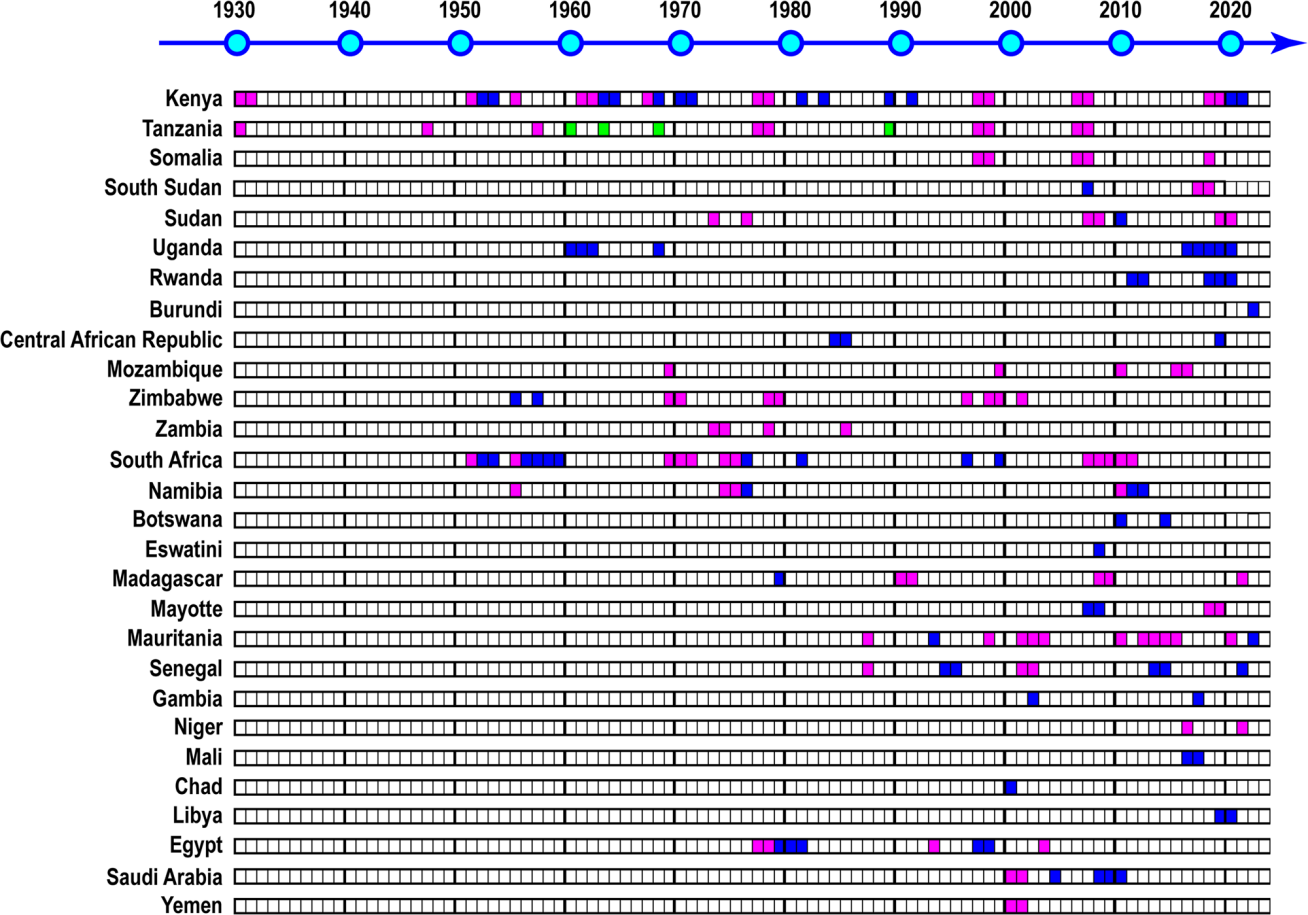
History of Rift Valley fever (RVF) outbreaks. The image depicts the historical RVF outbreaks. Blue represents isolated RVF outbreaks in animals or humans, red signifies major RVF outbreaks in animals, humans, or both, and green indicates limited information available on the outbreak.

The name RVF was given by Daubney et al. in 1930–31 after an outbreak in the Rift Valley, Kenya, which affected at least 3500 lambs, 1200 ewes, 50 cows, and 200 humans^9^. However, similar illnesses were reported in 1912 and 1926 in the same region^10^. RVFV was first isolated by Smithburn et al. from a pool of wild-caught mosquitoes from the uninhabited Semliki Forest in western Uganda in 1944^11^. Since then, there have been major RVF outbreaks in Kenya, including in 1951, 1967, 1977–78, 1997–98, 2006–07, and 2018–19^3,12^. Uganda has also experienced minor outbreaks in 1960–62, 1968, and 2016–20^13^. Other Eastern African countries, such as Tanzania, Somalia, Sudan, South Sudan, Rwanda, Burundi, Mozambique, Zimbabwe, and Zambia, have also suffered RVF outbreaks in the past several decades (Fig. 1)^14–16^. Phylogenetic analysis shows that the Kenya 1951 (Kenya 57, Rintoul) strain is closely related to the Uganda 1944 (Entebbe) strain, indicating that RVF was endemic to Kenya and Uganda in the 1940s–50s^17^. South Africa encountered its first RVF outbreak in 1950–51, which resulted in an estimated 100,000 deaths among sheep and 500,000 abortions among ewes^3^. Later, major RVF outbreaks occurred in 1974–76, 2007–08, and 2009–11. The South Africa 2009 strain clustered into the same phylogenetic clade as the Kenya 1951 or Zimbabwe 1974 (2373/74) strains, indicating that the virus likely originated from Kenya and/or Zimbabwe. Madagascar experienced its first RVF outbreak in 1990–91, with subsequent outbreaks in 2008–09 and 2021^3,18^. Mayotte, located close to Madagascar, also suffered RVF outbreaks in 2008 and 2018–19^14^. In Western Africa, RVFV was isolated from mosquitoes and humans in Senegal in the 1970s, but no major outbreak occurred until the 1980s^19^. The construction of the Diama Dam on the Senegal River in 1986 blocked the intrusion of saltwater and created more freshwater areas, which increased mosquito breeding, leading to a major outbreak in 1987 in southern Mauritania and northern Senegal^20^. RVF outbreaks have since occurred in Mauritania, Senegal, the Gambia, Mali, and Niger (Fig. 1)^14^. In 1977–78, a major RVF outbreak occurred in Egypt, leading to numerous deaths and abortions among livestock and an estimated 20,000–200,000 human infections, with morbidity in approximately 18,000 cases, mortality in 598 cases, and ocular disease in 800 cases^21^. RVF had never been reported in Northern Africa before this outbreak. After this major outbreak, RVF outbreaks occurred repeatedly in Egypt in 1993, 1997–98, and 2003. Despite the uncertain introduction route of the RVFV into Egypt, phylogenetic analysis indicates a genetic similarity between the Egyptian RVFV strains and those from Zimbabwe 1974 (2250/74) or Madagascar 1979 (MgH824) strain^22^, indicating the spread of the RVFV strain from the endemic region of sub-Saharan Africa. In 2000–01, the Arabian Peninsula experienced its first RVF outbreak in Saudi Arabia and Yemen, resulting in significant damage to the livestock industry. The outbreak caused approximately 8000–10,000 abortions and 40,000 deaths among livestock, including sheep, goats, and cattle. In addition, 883 human cases with 124 deaths were reported in Saudi Arabia, while Yemen reported 1328 human cases and 166 deaths^23^. The genomic sequence analysis revealed that the RVFV strain in the Arabian Peninsula, particularly in Saudi Arabia (Saudi10911 strain), was highly similar to the Kenya 1998 (Kenya00523) strain, suggesting its origin from the 1997–98 RVF outbreak across Kenya, Tanzania, and Somalia^22^.

## Virological characteristics of Rift Valley fever virus

RVFV is a member of the *Phlebovirus* genus within the *Phenuiviridae* family, which belongs to the *Bunyavirales* order. Its genome consists of three segments of RNA: Large (L), Medium (M), and Small (S). The S-segment has an ambi-sense polarity, encoding the nucleocapsid (N) gene in the negative-sense S RNA and the nonstructural S (NSs) gene in the positive-sense S RNA. The L- and M-segments are negative-sense RNA and encode the RNA-dependent RNA polymerase and the glycoprotein precursor proteins, respectively. The M-segment contains a single open reading frame (ORF), with different AUGs located in-frame at the N-terminal of the glycoprotein precursor proteins that can initiate polypeptide synthesis. The precursor protein generates the 78kD protein by co-translational cleavage when the first AUG is used^24^^,25^. If the second AUG is used, the precursor protein is co-translationally cleaved into the nonstructural NSm protein and two envelope glycoproteins (Gn and Gc)^25,26^. While the structural proteins (N, L, Gn, and Gc) are necessary for viral replication, the NSs, NSm, and 78kD proteins are not required for the replication of infectious RVFV.

RVFV entry into host cells involves interactions with different receptor molecules, including dendritic cell-specific intracellular adhesion molecule 3-grabbing nonintegrin (DC-SIGN), low-density lipoprotein receptor-related protein 1 (Lrp1), and heparan sulfate proteoglycan (HSPG)^27–29^. RVFV binds to DC-SIGN via high-mannose-type N-glycans present in both Gn and Gc^30^. HSPG binding also occurs via electrostatic contacts between negatively charged heparan sulfate groups and basic residues on the virus surface^29^. Lrp1, a protein with four ligand-binding regions (complement-like repeat clusters: CL_I_, CL_II_, CL_III_, and CL_IV_) interacts mainly with the RVFV Gn protein in the CL_IV_ cluster. Following attachment to host cell receptors, endocytic internalization of RVFV occurs, and acidification of late endosomes (pH 5.4 or below) leads to fusion of the Gc protein with the endosomal membrane, resulting in viral entry into the host cell cytoplasm^31^. The ribonucleocapsid (RNP) is released into the cytoplasm, and primary transcription occurs using L proteins from incoming virions to synthesize viral mRNA. Nascent mRNA is then used to synthesize viral proteins^32,33^. Switching from transcription to RNA replication can occur with newly synthesized N and L proteins in the cytoplasm. Glycoprotein precursor protein is synthesized in the endoplasmic reticulum (ER) and co-translationally cleaved into the Gn and Gc proteins. RVFV Gn proteins encode a Golgi retention signal, while RVFV Gc proteins have an ER retrieval signal^34^. Co-expressed Gn and Gc proteins interact with each other, localize to the Golgi complex, and recruit the RNP of L-, M-, and S-segment RNA for assembly^35^.

The NSs protein of the RVFV serves as a major virulence factor by acting as an antagonist of type-I interferon (IFN). In immunocompetent animals, RVFV lacking the NSs gene is highly attenuated. The RVFV NSs protein inhibits the upregulation of IFN-β mRNA but does not suppress transcription factors associated with the IFN-β promoter^36^. Two mechanisms by which NSs suppresses host transcriptional upregulation have been identified: inhibition of cellular RNA polymerase activities through the interruption of the assembly of the transcription factor IIH complex^37,38^ and epigenetic modification of the histone acetylation of chromosomal DNA through the recruitment of the Sin3-histone deacetylase complex^39^. The NSs-mediated post-translational degradation of PKR, a dsRNA-dependent protein kinase, allows for efficient viral protein synthesis under host transcriptional suppression^40^. Although pathogenic RVFV strains lacking the NSs gene cause lethal infections in mice lacking the IFN-A receptor or PKR and in immunocompetent C57BL/6 mice via intranasal inoculation, indicating that the NSs protein is not the sole virulence factor of RVFV^41,42^.

Both the 78kD and NSm proteins have been considered nonstructural proteins, leading to their alternatively being termed NSm1 and NSm2, respectively^43^. However, the 78kD protein can be incorporated into RVFV virions from infected mosquito C6/36 cells^44^ or Vero E6 cells^45^, indicating that it might be a structural protein. Recombinant RVFV, which has an optimized Kozak sequence for efficient expression of the 78kD protein, showed reduced infectivity in macrophage cell lines^45^, indicating potential interference with receptor binding due to the presence of the 78kD protein in the virion envelope. The 78kD and NSm genes are dispensable for lethal RVF disease in immunocompetent mice even via intraperitoneal administration^46,47^, while the 78kD protein plays a role in efficient RVFV dissemination from the midgut in *Aedes aegypti*^47^, indicating a function in mosquito vectors. The NSm protein can colocalize to the mitochondrial outer membrane and suppress the staurosporine-induced cleavage of caspases 8 and 9 in cultured cells such as Vero and 293 cells^48,49^, although the pathological role is still unclear.

## Host tropism of Rift Valley fever virus

RVFV has a varied host range with differing levels of susceptibility and disease outcomes^8,50–53^. Hosts with high mortality rates include lambs, calves, goat kids, mice, field voles (*Microtus agrestis*), Syrian hamsters (*Mesocricetus auratus*), and kittens. Severe disease with mortality is observed in sheep, cattle, goats, water buffaloes, humans, nonhuman primates (depending on species and condition), rats, gerbils, camels, ferrets, and gray squirrels (*Sciurus carolinensis*). Horses, cats, dogs, and nonhuman primates can be infected asymptomatically with viremia. Certain animals such as pigs, guinea pigs, mongooses (*Herpestes ichneumon*), hedgehogs, tortoises, frogs, chickens, pigeons, canaries, and parakeets are resistant to RVFV infection. RVFV targets dendritic cells early in the infection process, potentially affecting their maturation or migration^54,55^. Hepatocytes are also a primary target for acute RVF disease in many susceptible hosts. Lambs, mice, and hamsters can develop focal to diffuse hepatocellular necrosis following infection^56^. Splenic macrophages and other mononuclear phagocytic cells can also be infected, leading to necrotic red and white pulps in the spleen^57,58^. RVFV is highly neurotropic in many host species, including mice, rats, hamsters, gerbils, ferrets, nonhuman primates, and humans^59^. Highly susceptible lambs, mice, or hamsters can succumb to infection due to massive hepatic necrosis, while mice or hamsters surviving such liver injury eventually die from lethal encephalitis at a later stage of infection. Encephalitis is less common in livestock ruminants, although it has been reported in a 21-day-old calf^60^. Overall, RVFV can infect a wide range of hosts with varying levels of susceptibility and disease outcomes. It targets dendritic cells and hepatocytes early in the infection process, with subsequent neurotropic effects observed in many host species.

## Exploring the significance of genetic diversity in Rift Valley fever vaccine research

**Fig. 2:**
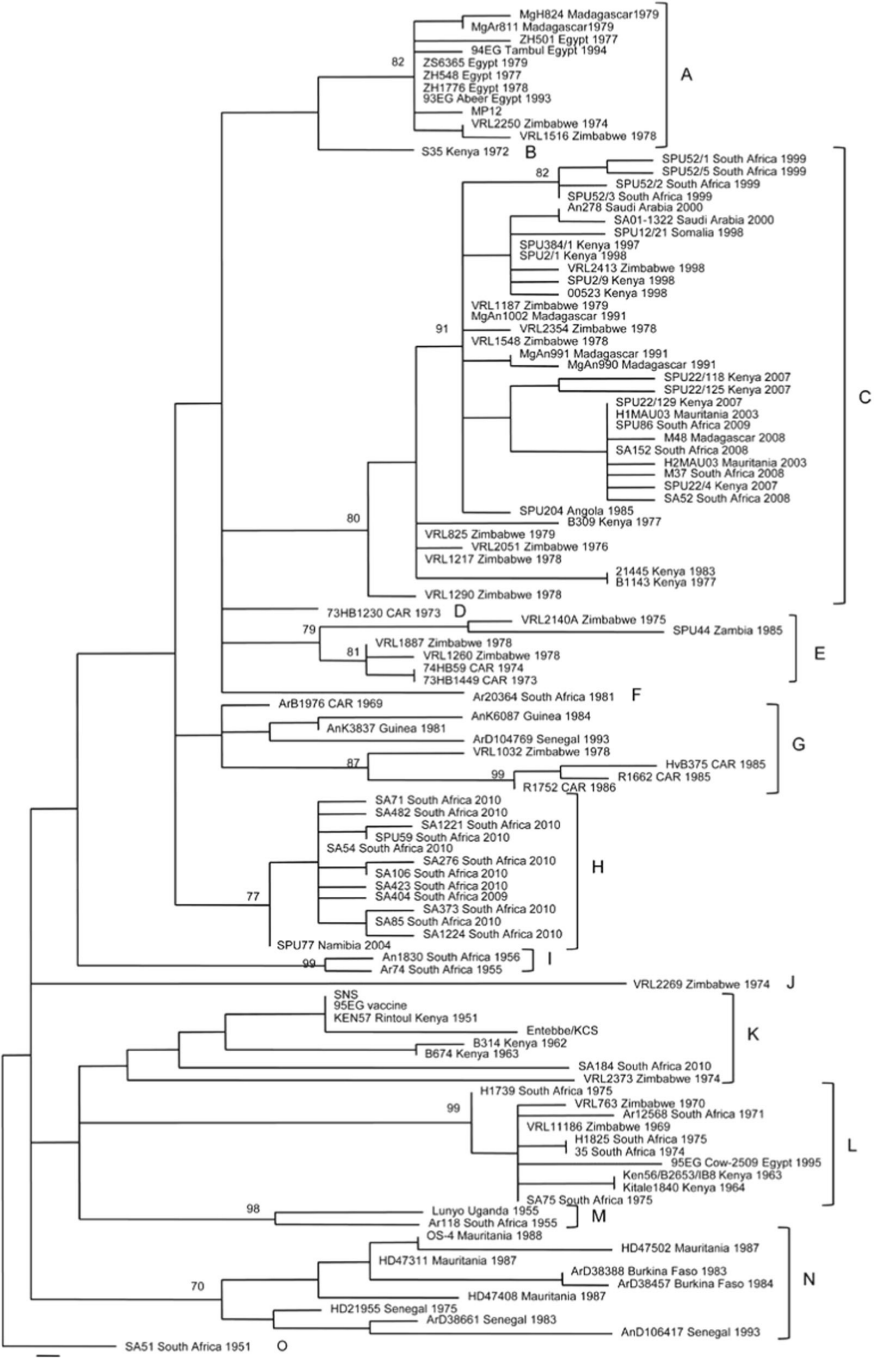
Genetic lineages of RVFV isolates. Genetic lineages of 111 RVFV isolates, determined using a 490-nt region of the Gn gene. The figure displays the names, countries of origin, and years of isolation. The bar represents 0.001 substitutions per site. This figure has been adopted and modified from Grobbelaar et al.^61^.

RVFV is characterized by a single serotype; however, its genetic diversity gives rise to 7 to 15 distinct genetic lineages (Fig. 2)^22,61^. In a study by Bird et al., 33 RVFV strains underwent comprehensive genome sequencing, uncovering variations at both nucleotide and amino acid levels. Specifically, differences were observed in the S segment (4% nucleotide, 1% amino acid), M segment (5% nucleotide, 2% amino acid), and L segment (4% nucleotide, 1% amino acid)^22^. Within endemic regions, genetic reassortment stands as a pivotal factor driving viral evolution^62^. Notably, it is worth mentioning that no instances of genetic reassortment have been reported between RVFV and other phlebovirus species up to the present^63^.

**Fig. 3:**
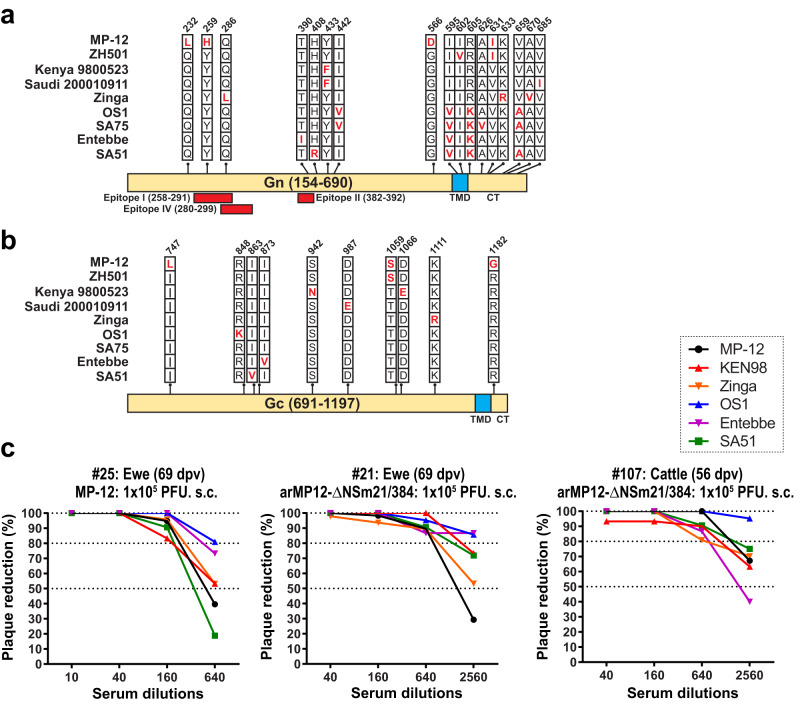
Cross-neutralization of Rift Valley fever virus strains by sheep or cattle sera vaccinated with MP-12 or arMP12-∆NSm21/384. A comparison was conducted on the amino acid sequences of Gn (**a**) or Gc (**b**) proteins among various strains of RVFV, including MP-12, ZH501, Kenya 9800523, Saudi Arabia 200010911, Zinga, OS1, SA75, Entebbe, and SA51. **c** The amino acid positions were determined based on the precursor protein from the first AUG start codon in the M-segment. In addition, the locations of neutralizing epitopes I, II, and IV^181^ were indicated. TMD refers to the transmembrane domain, while CT stands for cytoplasmic tail. In another experiment, sera obtained from pregnant ewes or cattle vaccinated with either MP-12 or rMP12-ΔNSm21/384^98,99^ were serially four-fold diluted. These diluted sera were then incubated with approximately 50 PFU of MP-12, wild-type Kenya 199800523, rZinga, wild-type OS1, wild-type Entebbe, or wild-type SA51 for the Plaque Reduction Neutralizing Test (PRNT). The percentage reduction in plaque numbers was calculated by comparing the plaque number with a normal control serum, which was set as 100%. This figure has been adopted from Ikegami et al.^58^.

The Gn and Gc proteins contain protective epitopes that stimulate the production of neutralizing antibodies. The variable amino acid sites in Gn and Gc among different strains are limited in number. To evaluate the efficacy, sera from vaccinated ewes or cattle with either MP-12 or arMP12-ΔNSm21/384 were tested using various RVFV strains: MP-12, wt Kenya 199800523, rZinga, wt OS1, wt Entebbe, and wt SA51^58^. As shown in Fig. 3, sera from vaccinated animals demonstrated a significant ability to reduce plaque formation by at least 80% for each RVFV strain. These findings indicate that vaccination with MP-12 or arMP12-ΔNSm21/384 can provide protection against RVFV strains from different genetic lineages due to the presence of cross-protective epitopes among different RVFV strains.

The vaccine development process involves the use of animal models with severe RVF diseases to assess the effectiveness of potential vaccines. Previous research has indicated variations in the virulence of different RVFV strains. Anderson et al. demonstrated that Egyptian strains (ZH501, ZH548, ZC3349, ZS6365, ZM657) exhibit greater pathogenicity compared to sub-Saharan RVFV strains (2051/76, 2269/74, Entebbe, Lunyo, SA51, SA75, B691) in Wistar Furth (WF) rats^64^. In a mouse model, the recombinant ZH501 (rZH501), SA51 (rSA51), and Zinga (rZinga) strains had median lethal doses (LD_50_) of 5.6, ≤1, and 10 PFU, respectively^58^. However, the relevance of this virulence observed in rodents to livestock animals or humans remains uncharacterized.

RVFV ZH501 strain has been assessed in nonhuman primate models. Rhesus macaques (*Macaca mulatta*) infected with ZH501 intravenously displayed asymptomatic cases (41%), mild febrile illnesses (41%), or severe hemorrhagic fever-like illnesses (18%)^65^. Subsequently, common marmosets (*Callithrix jacchus*) were found to be more susceptible to ZH501 challenge, with a survival rate of 0% via intranasal route and 50% via subcutaneous route^66^. However, another ZH501 challenge study using common marmosets (10^6.4^ PFU, s.c.) did not show any clinical signs of diseases except for transient viremia and an increase in the liver enzyme alanine aminotransferase (ALT)^67^. In a separate study, common marmosets were used to test the virulence of RVFV strains 35/74 (10^7^ TCID_50_, s.c. + i.m.) and 74HB59 (10^7^ TCID_50_, i.m.). In addition to transient viremia and an increase in ALT levels, 25% or 67% of infected animals exhibited clinical signs of disease, respectively^68^. The observed disparities in clinical findings among animal experiments involving common marmosets might stem from variations in viral strains, as well as differences in the sources or conditions of the animals used, which warrants additional attention in future studies.

Kroeker et al.^69^ presented an overview of different challenge models for RVF conducted in livestock ruminants. While the ZH501 strain was utilized in a sheep challenge model to study RVF, this specific model did not result in significant disease severity or mortality. To enhance the suitability of the sheep challenge model for RVF vaccine efficacy studies, researchers delved into using various RVFV strains, including Kenya-128B-15 (Ken06), recombinant strains 35/74 (South Africa), 56/74 (South Africa), AN1830 (South Africa), AR20368 (South Africa), or SA01-1322 (Saudi Arabia). Among these, the sheep challenge models involving the Ken06 strain administered subcutaneously or the recombinant 35/74 strain via intravenous injection displayed partial mortality, indicating a greater level of RVF disease severity compared to the ZH501 strain. Nonetheless, factors such as age, breed, route of inoculation, and viral passage history might influence the disease outcome.

## Classification of Rift Valley fever vaccines

The WHO released a draft of the Target Product Profile (TPP) for RVF vaccines in 2019, which categorizes them based on their intended purposes. These categories consist of: (i) emergency-use human RVF vaccines, (ii) human RVF vaccines for the protection of individuals at high risk, and (iii) RVF vaccines for sheep, cattle, goats, and dromedary camels. The TPP draft also outlines the minimum requirements for RVF vaccines in each category regarding protective immunity, shelf life, and safety. For instance, if RVF vaccines are intended for long-term protection in humans, they should provide a minimum of three years of immunity with either a single or up to two primary doses along with booster doses. These vaccines should have a shelf life of at least 12 months at −20 °C or 6 months at 2–8 °C. The TPP draft also emphasizes that during RVF outbreaks, no live-attenuated RVF vaccine should be administered to livestock or humans unless reassortment issues have been addressed. Regarding safety during pregnancy, the TPP draft states that live-attenuated RVF vaccines should not cause abortion or teratogenesis in pregnant animals, and the virus should not be transmitted vertically to the fetus.

Therefore, we have summarized the key characteristics of current and next-generation RVF vaccines in Table 1 based on the following criteria: (i) vaccine doses, (ii) potential for generating pathogenic reassortants, (iii) spread to mosquitoes, (iv) markers to differentiate infected from vaccinated animals, (v) safety during pregnancy in ewes, (vi) temperature stability, and (vii) cell substrates used in vaccine production. In terms of live-attenuated vaccines, two types, namely Smithburn and Clone 13, have been licensed for veterinary use in South Africa and other endemic countries^70^. Another live-attenuated vaccine, MP-12, has been conditionally licensed for use in livestock animals in the U.S. In the category of inactivated vaccines, formalin-inactivated Menya/Sheep/258 and binary ethylenimine-inactivated ZH501 vaccines, as well as a formalin-inactivated field RVFV strain, have been licensed and manufactured in Egypt and South Africa, respectively. It is important to note that there are currently no licensed RVF vaccines available for humans. However, two candidate vaccines, MP-12 and TSI-GSD-200, investigational new drugs, have undergone testing in humans in the U.S. In terms of the next-generation RVF vaccines, they can be classified into various categories, including live-attenuated vaccines, vector vaccines (such as those using adenovirus, poxvirus, alphavirus, Newcastle virus, equine herpes virus type 1, or rabies virus), subunit vaccines, RVFV single-cycle replicons, virus-like particles (VLPs), and DNA vaccines.

**Table 1:** Characteristics of current and next-generation Rift Valley fever vaccines.

Vaccines	Characteristics of vaccines^2^							In vivo studies for safety and immunogenicity^3^	Challenge strains^4^	References
	i	ii	iii	iv	v	vi	vii			
[Licensed vaccines]										
*Live-attenuated*										
Smithburn	A	E	C	C	C	B	D	Mice, Sheep, Cattle, Goat, Alpacas	a, c, m	^71–74,93,94,144^
Clone 13	A	D	C	B	C	B	B	Mice, Sheep, Cattle, Goat	a, c, l	^76,78,144–149^
MP-12^1^	A	D	C	C	C	B	A	Mice, Sheep, Cattle, Goat, Rhesus monkey, Alpacas	a	^87,98,150–157^
*Inactivated*										
Formalin-inactivated Menya/Sheep/258	C	A	A	B	A	B	?	?	?	^79^
Binary ethylenimine-inactivated ZH501 (ZH501-VSVRI)	C	A	A	B	A	B	D	?	?	^79^
Formalin-inactivated field RVFV strain	C	A	A	B	A	B	D	Sheep, Cattle	o	^80,158,159^
[Investigational New Drug vaccines]										
*Live-attenuated*										
MP-12	A	–	–	–	D	B	A	Mice, Sheep, Cattle, Goat, Rhesus monkey, Alpacas, Humans	a	^90,92^
*Inactivated*										
TSI-GSD-200	D	–	–	–	D	B	K	Sheep, cattle, goats, Humans	a	^85,160–162^
[Candidate vaccines]										
*Live-attenuated*										
DDVax^1^	A	C	B	B	D	B	B,D	Rats, Sheep, Ewes, Marmosets	a, b	^67,111,112,115^
RVFV-4s^1^	A	C	B	B	D	B	B,D	Mice, Lambs, Ewes, Calves, Goat kids, Marmosets	c, d	^68,106–110^
RIFTOVAX-LR and RIFTVAX-SR (Clone 13T)	A	D	C	B	B’	B	B	Sheep, cattle, goats	–	^116,118,163^
arMP12-∆NSm21/384	A	D	B	B	B	B	A,B	Mice, Lambs, Ewes, Calves, Goat kids	a	^48,98–101,164,165^
RVax-1	A	B	B	B	D	B	A,B	Mice	b	^97,102,104^
r2segMP12	A	C	B	B	D	B	B,D	Mice	–	^166^
*Adenovirus vector*										
ChAdOx1-GnGc^1^	B	A	A	A	A	A	C	Mice, Lambs, Ewes, Calves, Goat kids, Camels, humans	d, e	^119–123^
CAdVax-RVF	B	A	A	A	D	A	C	Mice	a	^167^
Ad5-GnGcopt	B	A	A	A	D	A	C	IFN-AR^-/-^ mice	k	^168^
Ad4-GnGc	B	A	A	A	D	A	C	IFN-AR^-/-^ mice	k	^126^
*Poxvirus vector*										
vCOGnGc	C	A	A	A	D	A	B	Mice, Baboon	a	^127^
MVA-GnGc	B	A	A	A	D	A	B	Mice	e	^128,169^
rLSDV-RVFV	C	A	A	A	D	A	B	Sheep	f	^129^
rKS1/RVFV	C	A	A	A	D	A	B	Mice, Sheep	m	^130^
*Alphavirus vector*										
REP91-RVF(M)	C	A	A	A	A	B	D	Mice	g	^133^
Rgrid-RVF(M)	C	A	A	A	A	B	D	Mice, Sheep	g	^133^
VEEV/Gn318(opt)	B	A	B	A	D	B	D	Mice	a	^132^
*Newcastle virus vector*										
NDFL-Gn	C	A	A	A	A	B	F	Cattle	–	^135^
NDFL-GnGc	B	A	A	A	A	B	F	Mice, Lambs	c, d	^141,170^
*Equine herpes virus type 1 vector*										
rH-Gn-Gc	C	A	A	A	D	A	E	Sheep	–	^136^
*Inactivated Rabies virus vector*										
rSRV9-eGn	D	A	A	A	A	B	D	Mice	–	^171^
*Subunit*										
RVFV GnGc subunit	C	A	A	A	A	B	G	Sheep, Cattle	h	^137–139^
GneS3	B	A	A	A	A	B	I	Mice, Lambs	c, d	^140,141^
RVFV-MPSP	C	A	A	A	A	B	H	Mice, Lambs	d	^142^
*RVFV replicon*										
RRP (NSR)	B	A	A	B	A	B	C,D	Mice, Sheep	c, d	^141,145,172,173^
VRP_RVF_	B	A	A	B	A	B	D	Mice	b	^174^
*RVFV VLP*										
VLP from rBac-N-G	C	A	A	B	A	B	G	Mice	–	^175^
VLP from S2 cells	C	A	A	A	A	B	I	Mice	c	^140^
VLP from 293T cells	B	A	A	B	A	B	J	Mice	i	^176^
*DNA vaccine*										
peGn	D	A	A	A	A	A	–	Mice	a, e	^177,178^
pCMV-Ub-N	C	A	A	B	A	A	–	IFN-AR^-/-^ mice	j	^179^
Gn-C3d	D	A	A	A	A	A	–	Mice	a	^134^
RVFV_-NSm_	D	A	A	A	A	A	–	Mice	a	^180^

^1^Candidate vaccines under concurrent evaluation for their human application.

MP-12 vaccine is conditionally licensed for veterinary use in the U.S.

^2^Characteristics of vaccines.

(i) Vaccine doses: A: Single dose without a booster, B: Single dose with booster(s), C: two primary doses with booster(s), D: three primary doses with booster(s).

(ii) Possibility of generating pathogenic reassortant(s) through vaccine-derived RNA segment(s): A: Not possible, B: Unlikely, C: Possible with one segment, D: Possible with two segments, E: Unknown due to uncharacterized attenuation mutations.

(iii) Spread to mosquito vectors: A: Unlikely, B: less probable, C: theoretically possible.

(iv) Markers for the differentiation of infected from vaccinated animals (DIVA): A: Absence of anti-N IgG, B: Absence of anti-NSs and/or anti-NSm IgG, C: no specific DIVA marker identified.

(v) Safety during pregnancy: A: Minimal concerns due to non-live or replicon vaccines, B: Considered safe if vaccinated at 42–53 gestation days (GD), C: Potential risk of abortion and/or fetal malformation if vaccinated between 42–53 GD, D: Safety during pregnancy has not been fully characterized, B’: Safety was confirmed in pregnant animals other than ewes.

(vi) Anticipated Temperature Stability: (A) Expected stability for a minimum of 1 month at 37 °C in lyophilized form, (B) Storage at 2–8 °C for a minimum of 6 months with optimized formulation in lyophilized form.

(vii) Cell or tissue substrates for vaccine preparation: (A) MRC-5, (B) Vero or its derivatives, (C) HEK293, (D) BHK derivatives, (E) RK-13, (F) Embryonated chicken eggs, (G) Sf9, (H) Sf9ET, (I) *Drosophila* Schneider S2, (J) HEK293T, (K) FhRL-2.

^3^Animal species or condition in vaccine studies are shown.

^4^Challenge RVFV strains: RVFV challenge strains used for testing protective efficacy of vaccine candidates: (a) ZH501, (b) rZH501, (c) M35/74, (d) r35/74, (e) 56/74, (f) AR 20368, (g) VRL 688/78, (h) Kenya-128B-15 (KEN06), (i) ZH548, (j) MP-12, (k) rMP-12, (l) Buffalo/99/MB/CER, (m) Smithburn pantropic or neurotropic, (o) other.

(–) Not applicable, (?) Little information available.

## Licensed live-attenuated Rift Valley fever vaccines for veterinary use: Smithburn, or Clone 13 vaccines

In the 1940s, Smithburn and colleagues demonstrated that repeated injections of the highly pathogenic Entebbe strain into the brains of mice (i.e., intracerebral [i.c.] passages) reduced its virulence after approximately 81 passages, resulting in the Smithburn neurotropic strain^71^. Further i.c. passages (~102nd) of this strain were used to develop a live-attenuated vaccine for veterinary use, which was made available in several endemic countries starting in the 1950s. Initially known as the “freeze-dried 10% mouse brain vaccine,” this vaccine was later modified in 1972 to allow for amplification of the vaccine strain without the need for mouse passages by using BHK-21 cells, resulting in the “modified live virus vaccine” (MLVV)^72^. Despite its efficacy and cost-effectiveness, the MLVV is not recommended for use in pregnant animals due to residual virulence that can lead to abortion and fetal malformations^73,74^. Nevertheless, the Smithburn vaccine has been distributed widely, with reported distributions of over 1 million doses in South Africa and Kenya (1951–1968), 6 million doses in Zimbabwe (1969–1970), 22 million doses in Namibia and South Africa (1974–1976), 4.2 million doses in South Africa, Egypt, and Israel (1977), 3 million doses in Zimbabwe (1978–1979), and 10 million doses in Saudi Arabia (2001)^61,75^.

The RVFV Clone 13 strain was plaque-isolated from a viral stock of the 74HB59 strain. The Clone 13 strain encodes a spontaneous 69% in-frame truncation of the NSs gene in the S-segment^76^. Since 2010, the Clone 13 vaccine has been registered as a veterinary RVF vaccine in several African countries, including South Africa, Namibia, Botswana, Zambia, and Mozambique. The lack of a functional NSs gene in the Clone 13 strain allows it to avoid suppressing host innate immunity, including the upregulation of type-I IFNs and IFN-stimulated genes, which contributes to the attenuation phenotype in healthy animals. However, the attenuation of the Clone 13 strain is solely due to the deletion of the NSs gene, and thus, acute lethal disease can be reproduced in mice that are knockout for type I IFN receptors (Ifnar-/-)^42^. The Clone 13 vaccine is highly immunogenic and is considered safe in pregnant animals, with a single dose being sufficient for immunization^77^. However, a safety evaluation of the Clone 13 vaccine using an overdose (1 × 10^6^ to 1 × 10^7^ median tissue culture infectious dose, TCID_50_) at 50 days of gestation resulted in the detection of virus in the placenta of vaccinated ewes, stillbirths, and fetal malformations^78^. This finding may be a potential concern regarding vaccination at early gestation. More than 28 million doses of the Clone 13 vaccine have been used, with at least 10 million doses being used in South Africa during the 2009–10 outbreaks^77^.

## Licensed inactivated Rift Valley fever vaccines for veterinary use

Three inactivated RVF vaccines have been developed specifically for veterinary use in regions where the disease is endemic. These vaccines are the BEI-inactivated RVF ZH501 vaccine (BEI-inactivated ZH501-VSVRI) developed by the Veterinary Serum and Vaccine Research Institute (VSVRI) in Egypt, the formalin-inactivated RVF Menya strain-based vaccine (formalin-inactivated Menya/Sheep/258) developed by VACSERA in Egypt, and the formalin-inactivated RVF vaccine derived from a field strain isolated from a cow in South Africa, manufactured by the Onderstepoort Biological Product (OBP) in South Africa^79–81^. The BEI-inactivated ZH501-VSVRI vaccine and formalin-inactivated Menya/Sheep/258 vaccine are adjuvanted with alum, making them safer options compared to the Smithburn vaccine, which is also manufactured at VSVRI^79^. The inactivated RVF vaccine from OBP is also formulated with aluminum hydroxide gel as adjuvant. It is recommended for cattle, sheep, and goats at any age regardless of the stage of pregnancy or lactation. However, for calves and lambs, it is only recommended after they reach the age of six months. While live-attenuated RVF vaccines provide long-lasting protection with a single dose, inactivated RVF vaccines require two primary doses and annual booster doses to maintain protective immunity over an extended period. Nevertheless, the use of inactivated RVF vaccines in pregnant or infant animals poses fewer safety concerns compared to live-attenuated vaccines.

## Formalin-inactivated Rift Valley fever candidate vaccines for humans: TSI-GSD-200

Although several RVF vaccines are available for veterinary use in endemic countries, there are no licensed RVF vaccines available for human use. Therefore, it is crucial to develop effective RVF vaccines for both animals and humans. The initial batch of the formalin-inactivated RVF vaccine (designated as National Drug Biological Research-103 or NDBR-103) was produced using a production seed derived from viremic mouse serum that contained the Entebbe strain, using primary African green monkey kidney cells^82,83^. Its safety and immunogenicity were evaluated in several thousand people, including United Nations (UN) Expeditionary Forces soldiers in the Sinai and high-risk laboratory personnel^82^. However, NDBR-103 was replaced by TSI-GSD-200, which was manufactured using fetal rhesus monkey lung cells (FRhL-2) at the Salk Institute Government Service Division (Swiftwater, PA)^82–84^. The TSI-GSD-200 vaccine is an Investigational New Drug (IND) vaccine that has been given to high-risk personnel, including laboratory workers, in the U.S. The TSI-GSD-200 vaccination regimen consists of three primary vaccinations at 0, 10, and 28 days. The half-life of the plaque reduction neutralizing test 80 (PRNT_80_) titer of ≥1:40 after the three primary doses is shown to be 315 days^85^. Thus, the TSI-GSD-200 vaccine requires at least one booster vaccination after primary doses to maintain protective immunity. The increasing regulation of select agents and validation of inactivation may hamper further production of the same RVF vaccine using a high-containment facility. Therefore, there is an urgent need to develop new RVF vaccines that can be manufactured safely and effectively, as the prevention of RVF in humans is of utmost importance due to the high morbidity associated with the disease.

## Live-attenuated Rift Valley fever candidate vaccine: MP-12

In the 1980s, a live-attenuated vaccine candidate for RVF, called MP-12, was developed for animal and human use^86^. The vaccine was produced by subjecting the pathogenic ZH548 strain to 12 sequential plaque passages in MRC-5 cells, in the presence of the chemical mutagen 5-fluorouracil^86,87^. The resulting MP-12 vaccine carries a total of 23 mutations in the viral genome, distributed in the S, M, and L segments. Notably, two amino acid changes in the envelope proteins of the M-segment (Gn-Y259H and Gc-R1182G) are major contributors to the attenuated phenotype of the MP-12 strain^88^. In addition, the MP-12 strain has a weak temperature-sensitive phenotype, which restricts its replication at temperatures above 38 °C^89^. The MP-12 vaccine was produced at the Salk Institute Government Service Division (TSI-GSD-223) for preclinical and clinical studies^87^. Animal studies demonstrated that a single dose of MP-12 vaccine could induce protective neutralizing antibodies in mice, sheep, cattle, goats, and rhesus macaques, with a minimal protective neutralizing antibody titer estimated at 1:5 (PRNT_80_) in mice^90^. In 2013, the MP-12 vaccine was conditionally licensed for veterinary use in the U.S. Meanwhile, clinical trials in human volunteers evaluated the safety and immunogenicity of the vaccine. These trials revealed that a single intramuscular injection of MP-12 vaccine could induce a peak geometric PRNT_80_ titer of 1:277 with a 10^4.4^ PFU dose, and protection could be maintained for at least 1 year with a PRNT_80_ titer of at least 1:20 in most vaccinees. Mild to moderate adverse effects were observed^91,92^, including headache, malaise, dizziness, chills, vomiting, and fever, while no major adverse effects were reported. Transient increases in serum enzymes such as alanine transferase (ALT), aspartate aminotransferase (AST), creatine phosphokinase (CPK), and lactate dehydrogenase (LDH) were observed in some vaccinees, but their relevance to vaccination was unclear.

## Rationale for next-generation RVF vaccine candidates

**Fig. 4:**
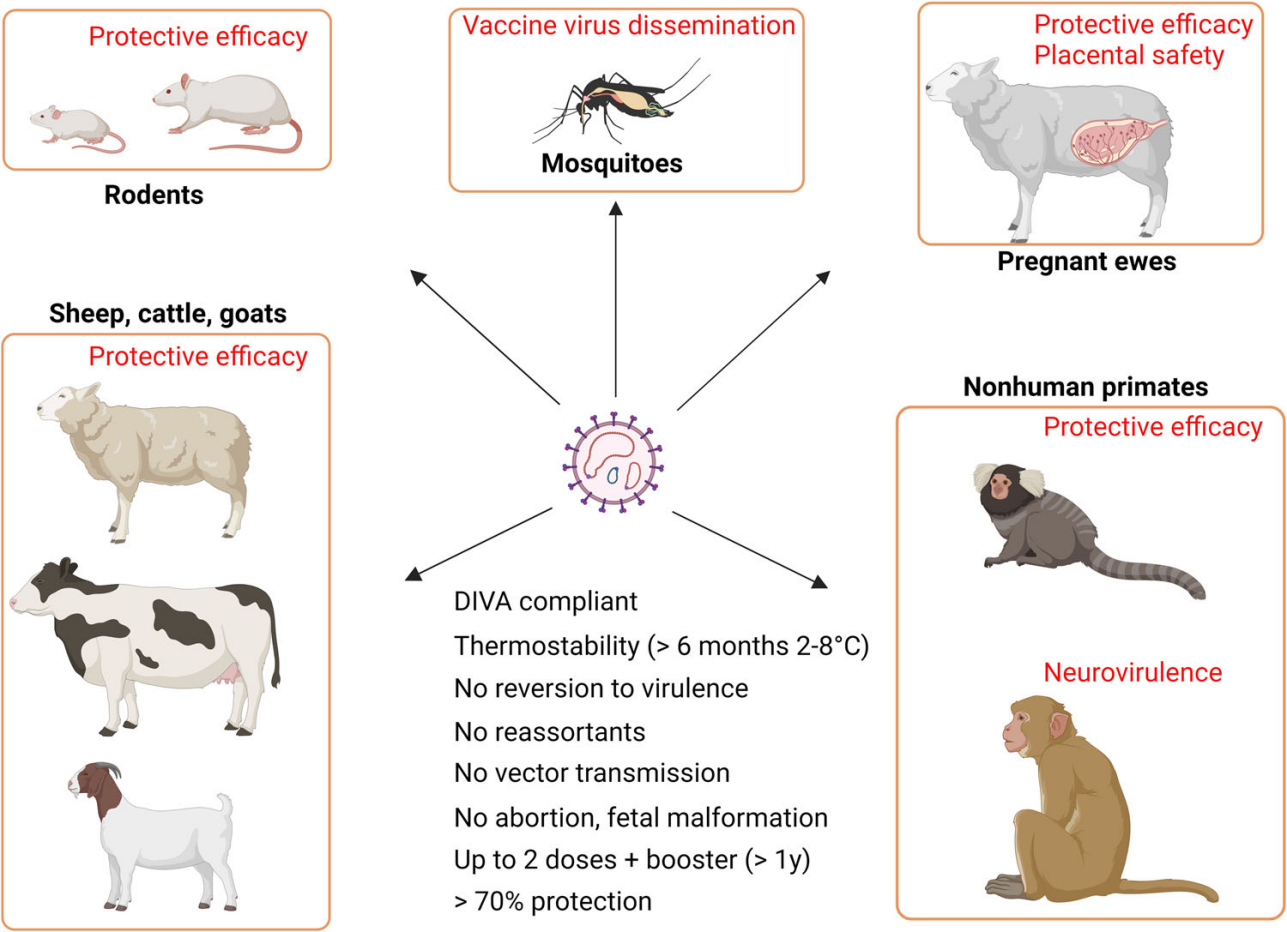
Schematic of the effective vaccine design for Rift Valley fever (RVF) vaccine. Testing of RVF vaccines includes evaluation for safety, immunogenicity, and protective efficacy in relevant animal models, as well as assessment of vaccine virus dissemination in mosquitoes. Meeting the key requirements proposed by the World Health Organization for RVF vaccine standardization is essential to achieve the Target Product Profile for RVF vaccines. Images were created with BioRender.com.

Live-attenuated RVF vaccines are known to be more effective than inactivated vaccines, providing cost-effective protection against RVFV infection. The live-attenuated vaccines replicate the virus strain, mimicking natural infection, and induce humoral and cellular immunity against various viral epitopes. However, there are several potential drawbacks to the use of live-attenuated RVF vaccines, including residual virulence in pregnant and newborn ruminants, a lack of effective DIVA markers, limited attenuation in immunocompromised animals, and the possibility of vaccine strain spill over infection in mosquitoes (Fig. 4).

The Smithburn vaccine, for example, retains residual virulence, which can lead to spontaneous abortions, stillbirths, or fetal death in pregnant ewes, cows, or does, as well as lethal viral meningoencephalitis in young and adult alpacas^73,74,93,94^. Similarly, the Clone 13 vaccine has a potential risk of infecting the placenta and fetuses, leading to stillbirth, fetal malformation, or viral RNA detection in fetuses^78^. In non-endemic countries, it is crucial to develop DIVA markers to differentiate between infected and vaccinated animals. Unfortunately, current live-attenuated vaccines such as the Smithburn and MP-12 vaccines do not encode any DIVA markers, while the Clone 13 vaccine’s DIVA marker (partial truncation of the NSs gene) is poorly immunogenic, making it a less sensitive marker^95^. Meanwhile, whole inactivated RVFV vaccines require high containment facilities for production and multiple primary and booster doses to maintain protective immunity, limiting their practicality. Given these drawbacks, researchers study next-generation RVF vaccines that could potentially overcome these limitations by improving safety or immunogenicity.

## Next-generation MP-12 vaccines: arMP12-∆NSm21/384, RVax-1, or r2segMP12

The MP-12 vaccine has been genetically modified via reverse genetics to produce the next-generation candidate vaccines^96^. The RVFV infectious clones have been rescued using BHK-derived cells such as BHK/T7-9 or BSR T7/5, but further safety validations are required to exclude their tumorigenicity and the presence of adventitious agents. To address this issue, a next-generation reverse genetics system was developed for the direct recovery of infectious clones from Vero cells, which can be applied to vaccine cell substrates for vaccine production purposes^97^.

One of the recombinant MP-12 strain variants, arMP12-∆NSm21/384, encodes an in-frame deletion of the 78kD and NSm genes in the M-segment ORF^48^. This deletion has the potential advantage of minimal vaccine virus dissemination in mosquito vectors and the application of 78kD or NSm as DIVA markers. The immunogenicity and protective efficacy of arMP12-∆NSm21/384 were found to be equivalent to those of the original MP-12 vaccine in pregnant ewes, lambs, and calves^98–100^. arMP12-∆NSm21/384 (1 × 10^5^ TCID_50_) did not cause any fetal malformation in ewes vaccinated at 43 to 150 gestation days, while three out of 17 (18%) ewes vaccinated at 7 to 34 gestation days had fetal malformations in the tail or forelimb, although RVFV RNA was not detected in the affected lambs^101^. Thus, further validation of the vaccine safety of the arMP12-∆NSm21/384 candidate vaccine in pregnant animals is required to determine its suitability for use in livestock ruminants.

Another variant, rMP12-GM50, encodes 584 silent mutations located every 50 nucleotides across the N, NSs, M, and L ORFs in the backbone of the MP-12 strain. These silent mutations were designed not to deoptimize codon usage or codon pair bias to maintain efficient virus replication in mammalian cells. In outbred mice infected with one of three pathogenic reassortant ZH501 strains encoding the rMP12-GM50 L-, M-, or S-segments, survival rates were 90%, 50%, and 30%, respectively, while those infected with a reassortant ZH501 strain encoding the L-, M-, or S-segments of the parental MP-12 strain showed survival rates of only 20%, 40%, and 0%, respectively^88,102^. A single dose of rMP12-GM50 induced 1:160–1:2560 PRNT_80_ titers at 42 dpv in CD1 mice and protected against pathogenic rZH501 challenge^102^. The rMP12-GM50 strain exhibits a unique silent mutation pattern that can aid in the detection of genetic reassortants or recombination events with other RVFV strains^103^. In addition, attenuations of RVFV reassortant strains that encode the L- or S-segments of the rMP12-GM50 strain can also be detected using this mutation pattern. Therefore, further genetic modifications were made to the rMP12-GM50 strain to create the third-generation MP-12 candidate vaccine, RVax-1. The RVax-1 vaccine is a variant of rMP12-GM50 that includes an in-frame deletion of the 78kD and NSm genes, similar to the deletion found in the arMP12-∆NSm21/384 strain. RVax-1 was found to replicate as efficiently as the rMP-12 strain in Vero or MRC-5 cells, and the 566 unique silent mutations in the RVax-1 genome remained genetically stable over 10 viral passages in Vero cells^104^ When *Aedes aegypti* mosquitoes were fed RVax-1 orally, the virus showed inefficient dissemination outside of the midgut, unlike rMP-12, which demonstrated systemic viral dissemination by 14 days post-infection (dpi) in various tissues, including the midgut epithelial cells, fat bodies, cerebral ganglions, ommatidia, and salivary glands^104^. In C57BL/6 mice, a single dose of RVax-1 or rMP-12 led to low-level viremia (100–200 PFU/ml) at 3 days post-vaccination (dpv), while inducing PRNT_80_ neutralizing antibodies at an equivalent level to control rMP-12 by 14 dpv and these antibodies persisted for at least 98 dpv^104^. Further characterization of the RVax-1 candidate vaccine in terms of its immunogenicity and attenuation properties is required to support its development as a vaccine.

The r2segMP12 vaccine candidate modifies the S-segment to encode the Gn and Gc ORF instead of the NSs gene, resulting in a virus genome consisting of only the S- and L-segments^105^. Vaccinating CD-1 outbred mice with 1 × 10^5^ PFU of this candidate vaccine yielded PRNT_50_ titers ranging from 1:20 to 1:320. This vaccine candidate’s lack of the NSs and 78kD/NSm genes, along with genetic rearrangement from three segments to two segments, suggests a superior attenuation profile compared to arMP12-∆NSm21/384 or RVax-1. Further optimization of the vaccination regimen to enhance immunogenicity would support its application in both humans and animals.

## Live-attenuated RVF vaccine candidate: RVFV-4s

The live-attenuated RVFV-4s vaccine was created by splitting the M-segment of the RVFV into two segments encoding NSm-Gn and Gc^106^. This genetic modification did not impact virus replication efficiency in BSR-T7/5 cells, but it led to a significant reduction in virus production in *Aedes albopictus* C6/36 cells. In vaccinated mice, RVFV-4s was found to be avirulent, with no detectable viral RNA in the liver or brain from 1 to 11 dpv. Two different backbones were used to generate RVFV-4s: the Clone 13 strain backbone (hRVFV-4s) or the 35/74 strain backbone with a deletion of the NSs gene in the S-segment (vRVFV-4s). RVFV-4s was found to be effective in inducing neutralizing antibodies against RVFV with a single intramuscular dose in various animal species, including lambs, calves, goat kids, pregnant ewes, and common marmosets^68,107–110^. In addition, RVFV-4s was shown to provide protection against pathogenic RVFV challenge in lambs, calves, goat kids, and pregnant ewes between 101–105 days post-vaccination^109,110^. In terms of safety, RVFV-4s was administered to pregnant ewes at 44 days of gestation (1 × 10^7^ TCID_50_), and no clinical signs of disease or detectable viral RNA were observed in the ewes or fetuses^108^, indicating that the vaccine is safe for use in pregnant ewes. Further research is needed to fully characterize RVFV-4s, particularly in terms of long-term protective efficacy, neurovirulence, and optimization of vaccine stability under ambient and cold temperatures. These findings highlight the potential of RVFV-4s as a candidate vaccine for both veterinary and human applications.

## Live-attenuated RVF vaccine candidate: DDVax

The DDVax vaccine is a live-attenuated vaccine developed from the recombinant ZH501 strain, achieved by deleting both the NSs and the 78kD/NSm genes^111,112^. The NSs gene deletion reduces the vaccine’s virulence in immunocompetent animals while robustly stimulating innate immune responses. The vaccine’s immunogenicity and protective efficacy against pathogenic RVFV challenge have been demonstrated in rats, pregnant ewes at 42 days of gestation, and common marmosets^67,111,112^. To assess vaccine safety, 20 vaccinated ewes at 42 days of gestation were monitored until they delivered neonates, and it was found that vaccination with DDVax did not lead to detectable viral RNA in the sera^112^. Each vaccinated ewe delivered at least one healthy lamb without any detectable viral RNA in the blood, brain, liver, and spleen. Although vaccinated ewes did not have anti-NSs or anti-NSm antibodies detectable by the enzyme-linked immunosorbent assay (ELISA), they showed the seroconversion of anti-NSs or anti-NSm antibodies after pathogenic RVFV challenge. This supports the concept of DDVax as a DIVA-capable RVF vaccine candidate. The DDVax candidate vaccine shows superior performance in reducing viral transmission via vector mosquitoes compared to Clone 13 and MP-12 vaccines. Experimental oral infections of *Aedes aegypti* mosquitoes with the DDVax candidate vaccine demonstrated inefficient viral dissemination from the midgut to the salivary glands or ovaries^113–115^. Further characterization of the DDVax vaccine is required to support its future application in animals and humans. These include evaluating its long-term protective efficacy, neurovirulence, and optimizing its stability. Nonetheless, the DDVax vaccine is one of promising candidates for preventing RVFV infection in both animals and humans.

## Live-attenuated RVF vaccine candidate: Clone 13T

To enhance the thermostability of the veterinary Clone 13 vaccine^116^, researchers undertook a process of isolating the Clone 13 virus from Vero cells cultured at 56 °C. This isolation was completed by a series of three heating and selection cycles^117,118^. The outcome of this process was the emergence of a new virus variant termed Clone 13T. Notably, Clone 13T exhibited improved thermostability at both 37 °C and 45 °C when compared to the original Clone 13 virus. Clone 13T maintained its stability at 4 °C for a span of over 18 months in a lyophilized state^117^. This candidate vaccine has undergone registration under the names RIFTOVAX-LR and RIFTOVAX-SR in Morocco, while it has undergone the immunogenicity testing on ruminant livestock in the regions of Senegal and Mali^70^.

## Chimpanzee adenovirus vector RVF vaccine candidate: ChAdOx1 RVF

The early region E1-and E3-deleted replication-deficient chimpanzee adenovirus vector (ChAdOx1) encoding RVFV MP-12 GnGc proteins was generated as a candidate vaccine (ChAdOx1-GnGc or ChAdOx1 RVF). This candidate vaccine lacks expression of the highly antigenic RVFV N protein, making it useful for DIVA in veterinary vaccination. Administration of a single dose of ChAdOx1-GnGc with or without a saponin-based Matrix-M or Matrix-Q adjuvant induced neutralizing antibodies in various animal species, including mice, ewes, lambs, goat does, goat kids, calves, and camels^119–121^. All animals demonstrated protective efficacy against pathogenic RVFV challenge, except for some vaccinated goats, where RVFV RNA or infectious RVFV was detected in the placentomes^119,120^. Pregnant ewes, on the other hand, showed complete protection against RVFV challenge^119^. To improve the thermostability of ChAdOx1-GnGc, it was dried on a Whatman S14 glass fiber (GF) membrane containing a mixture of trehalose and sucrose^122^. The dried vaccine was stored at different temperatures for up to six months and reconstituted with buffer solution. The vaccine reconstituted after storage at 25, 37, or 45 °C in the GF membrane for six months was found to elicit comparable neutralizing titers in vaccinated cattle. However, vaccines stored at 55 °C failed to induce detectable antibody responses. This thermostabilized ChAdOx1-GnGc vaccine will be particularly useful in regions where maintaining a cold chain is challenging. A phase 1, open-label, dose escalation trial was conducted in the United Kingdom from June 2021 to January 2023 to evaluate the safety and immunogenicity of the ChAdOx1 RVF vaccine in healthy adults aged 18–50 years^123^. Among the groups vaccinated intramuscularly with a single dose of 5.0 × 10^9^ (*n* = 3), 2.5 × 10^10^ (*n* = 6), or 5.0 × 10^10^ (*n* = 6) virus particles of the ChAdOx1 RVF vaccine, the highest-dose group elicited high levels of neutralizing antibodies that peaked at 28 dpv and persisted until 84 dpv. The vaccine was generally well-tolerated, with mild to moderate adverse events reported in some participants. The ChAdOx1 RVF vaccine is now being investigated further in a phase 1b trial in healthy adults in Uganda (NCT04672824).

## Various viral vector vaccine candidates for RVF

Vector vaccines can be classified into three types: live vectors, single-cycle replicons, or replication-deficient vectors. Compared to classical live-attenuated vaccines, vector vaccines have unique properties, such as thermostability or scalability, and are also DIVA compliant. However, vector vaccines also have potential drawbacks. One concern is the stability of the inserted protective antigens, which may affect the vaccine’s effectiveness. Another concern is reduced immunogenicity in hosts with pre-existing immunity due to prior exposure to the vector.

A specific replication-defective complex adenovirus (CAdVax) vector system has been developed using a modified human adenovirus type 5 vector backbone with deletions in E1, E3, and almost all E4 ORFs, except for ORF6^124^. The CAdVax-RVF vaccine encodes codon-optimized Gn and Gc derived from the RVFV MP-12 strain under the human CD4 signal peptide. Another candidate vaccine, Ad5-GnGcopt, was generated using the commercially available AdMax adenovirus system^125^. This vaccine is an E1/E3-deleted, replication-deficient human adenovirus type 5 vector encoding codon-optimized Gn and Gc derived from the RVFV MP-12 strain under the tissue plasminogen activator signal peptide. Studies have shown that pre-existing neutralizing antibodies against human adenovirus type 5 may reduce the specific humoral and cellular immune responses to the Ad5-based vaccines. However, optimizations of doses or administration routes for vaccinations might overcome the impact of pre-existing immunity against Ad5. In contrast, a replication-competent human adenovirus type 4 was generated to express RVFV Gn and Gc antigens^126^. This Ad4-GnGc candidate vaccine was not affected by the pre-existing immunity against Ad5 and was found to induce robust protective immunity in a mouse model.

Poxvirus vectors are an attractive option for vaccine development due to their thermostability and DIVA compliance. These vectors allow for the expression of viral antigens from highly attenuated backbone vectors, including the Copenhagen strain vector encoding RVFV Gn and Gc (vCOGnGc)^127^, modified vaccinia virus Ankara strain vector encoding RVFV Gn and Gc (MVA-GnGc)^128^, a recombinant lumpy skin disease virus vector encoding RVFV Gn and Gc (rLSDV-RVFV)^129^, or a recombinant capripoxvirus vector encoding RVFV Gn and Gc (rKS1/RVFV)^130^. Furthermore, the rLSDV-RVFV or rKS1/RVFV vectors confer dual protective immunity against both RVF and sheep pox viruses, while the MVA-GnGc has been modified to co-express bluetongue virus antigens, providing dual protection against RVF and bluetongue^131^.

Alphavirus vectors, such as Sindbis virus (SINV) or Venezuelan Equine Encephalitis virus (VEEV), are also promising candidates for RVF vaccines due to their ability to replicate robustly in mammalian cells and express RVFV Gn antigens. Alphavirus replicon vectors do not encode alphavirus structural proteins in their genome, which can reduce host anti-vector immune responses. A few studies have evaluated the immunogenicity and protective efficacy of alphavirus vector vaccines: SINV replicon vectors (REP91-RVF(M) or Rgrid-RVF(M)) in mice or sheep, VEEV replicon vectors (e.g., VEErep/spGn) in mice, or chimeric VEEV vectors [e.g., VEE/Gn318(opt)] in mice^132–134^. RVFV Gn expression was found to affect the efficient production of VEEV replicons in cultured cells, which required extensive optimization of the VEEV vector^132^. Overall, these findings suggest that both poxvirus and alphavirus vectors hold good potential for the development of effective RVF vaccines.

The Newcastle disease virus (NDV) LaSota strain is a safe option for vaccine applications as it is non-pathogenic to both mammals and birds. This is due to the fact that NDV is not a natural host for these species, resulting in low pre-existing immunity against NDV in humans and livestock ruminants. The scalable production of NDV vector vaccines can be achieved using embryonated eggs, similar to the production of influenza vaccines. In animal models, live NDV vector vaccines for RVF, including NDFL-Gn and NDFL-GnGc, have been tested for their immunogenicity and protective efficacy^135^. In addition, a live Equine herpesvirus type 1 (EHV-1) RacH strain vector vaccine for RVF, rH_Gn-Gc, has been generated and studied for immunogenicity in sheep^136^. EHV-1 has a broad cell tropism and lacks pre-existing immunity in non-equine animals. Sheep vaccinated with rH_Gn-Gc demonstrated high potency as a vector vaccine for RVF, with PRNT_50_ titers reaching up to 1:320.

## Single-cycle RVFV replicon vaccine candidates: NSR-Gn or VRP_RVF_

Nonspreading single-cycle replicon RVF vaccines (NSR-Gn) encodes Gn in place of NSs gene but lack the M-segment RNA. Due to a lack of Gc in the genome, resulting virions do not create infectious virus particles after the first-round replication. Meanwhile, RVFV replicon particles (VRP_RVF_) encodes the green fluorescent protein in place of NSs gene but lack the M-segment RNA in the ZH501 strain backbone. The VRP_RVF_ does not carry Gn and Gc genes, which prevents viral spread after the first-round replication. The neutralizing antibody titers reached 1:40–1:320 at 28 dpv with single dose VRP_RVF_ vaccination in mice. Both NSR-Gn and VRP_RVF_ can confer protective immunity in vaccinated animals with little adverse effects.

## Subunit vaccine candidates: GneS3, RVFV GnGc subunit vaccine, or MPSP-Gn head vaccine

A subunit vaccine candidate for RVF has been developed that is DIVA compliant and can be produced without the need for high containment facilities. This vaccine induces a moderate level of immunogenicity, but the addition of an adjuvant and optimization of vaccination regimens can lead to prolonged protective immunity in vaccinated animals.

One such subunit vaccine candidate is the RVFV GnGc subunit vaccine, which contains the ZH548 Gn ectodomain and the full-length ZH548 Gc protein formulated in montanide ISA25 water-in-oil adjuvant. These Gn and Gc proteins were purified from Sf9 cells infected with recombinant baculoviruses encoding Gn ectodomain or Gc^137^. When tested in vaccinated sheep, this vaccine induced PRNT_80_ neutralizing antibody titers of 1:10–1:160 at 14 dpv and >1:1280 at 28 dpv (7 days post the second booster). Importantly, vaccinated animals did not produce anti-N antibodies, which can be used for DIVA purposes. When challenged with the pathogenic RVFV Kenya-128B-15 (Ken06) strain at 14 days post the second booster, all vaccinated sheep survived and were protected from viremia or clinical signs of disease^138^. Similarly, calves vaccinated with the RVFV GnGc subunit vaccine showed 100% protection against the Ken06 strain challenge at 35 dpv, regardless of whether they received a booster dose at 21 dpv or not^139^.

Another subunit vaccine candidate, designated as GneS3, is formulated in Stimune (a water-in-oil adjuvant) with RVFV Gn ectodomain purified from Drosophila Schneider (S2) cells^140^. Lambs vaccinated with GneS3 raised neutralizing antibodies at 19 dpv and were protected from viremia and fever after challenge with the pathogenic recombinant 35/74 strain. Since neutralizing antibody titers were increased after a challenge with pathogenic RVFV, it was indicated that vaccinated lambs allowed some replications of the challenged virus^141^.

A third vaccine candidate is based on the N-terminal 314 amino acids of RVFV Gn (head domain) conjugated to SpyTag-displaying multimeric protein scaffold particles (MPSPs)^142^. Mice or lambs vaccinated twice with MPSP-Gn head vaccine candidates were protected from pathogenic RVFV challenges and showed neutralizing antibody titers of 1:1000 or higher after the second vaccination. These MPSP-Gn head vaccine candidates self-assemble into different sizes of nanoparticles, providing flexibility in vaccine design.

In summary, subunit RVFV vaccine candidates offer a safe and effective alternative to live-attenuated vaccines, and their production is relatively simple and cost-effective. With the addition of adjuvants and optimization of vaccination regimens, these vaccines can confer prolonged protective immunity in vaccinated animals.

## Future perspectives

There have been many reported RVF candidate vaccines including whole inactivated vaccines, live-attenuated vaccines, vector vaccines, replicon vaccines, subunit vaccine, virus-like particle vaccines, DNA vaccines^143^. Currently, there are no licensed RVF vaccines for human use. However, formalin-inactivated TSI-GSD-200 and live-attenuated MP-12 vaccines have undergone clinical trials in the past. The Coalition for Epidemic Preparedness Innovations (CEPI) has provided funding to support accelerated phase I and II clinical trials for RVFV-4s and DDVax candidate vaccines. Promising RVF candidate vaccines, such as hRVFV-4s and ChAdOx1 RVF, have entered phase I clinical trials in 2022. Further safety and immunogenicity studies for promising RVF candidate vaccines should be warranted for reducing future RVF threats.

RVF is a severe zoonotic disease that poses a significant threat to both human and livestock health in endemic regions. To reduce the likelihood of future devastating RVF outbreaks, effective RVF vaccination programs using appropriate vaccines for susceptible livestock animals are crucial. The WHO has outlined the minimally acceptable profiles of target RVF vaccines. These include protective immunity, shelf life, and safety. For future RVF vaccines, superior thermostability and environmental safety, as well as safety in pregnant animals or immunocompromised humans, should be considered, particularly for immunization during RVF outbreaks. In addition, an effective vaccination strategy using an RVF vaccine with strong vaccine immunogenicity is essential for eradicating RVF from endemic regions. Promising RVF candidate vaccines require further safety and immunogenicity studies to reduce future RVF threats.

### Reporting summary

Further information on research design is available in the Nature Research Reporting Summary linked to this article.

### Supplementary information

Reporting Summary
